# Investigating Multiple Candidate Genes and Nutrients in the Folate Metabolism Pathway to Detect Genetic and Nutritional Risk Factors for Lung Cancer

**DOI:** 10.1371/journal.pone.0053475

**Published:** 2013-01-23

**Authors:** Michael D. Swartz, Christine B. Peterson, Philip J. Lupo, Xifeng Wu, Michele R. Forman, Margaret R. Spitz, Ladia M. Hernandez, Marina Vannucci, Sanjay Shete

**Affiliations:** 1 Division of Biostatistics, University of Texas School of Public Health, Houston, Texas, United States of America; 2 Department of Statistics, Rice University, Houston, Texas, United States of America; 3 Human Genetics Center, The University of Texas School of Public Health, Houston, Texas, United States of America; 4 Department of Epidemiology, The University of Texas MD Anderson Cancer Center, Houston, Texas, United States of America; 5 Dan L. Duncan Cancer Center, Baylor College of Medicine, Houston, Texas, United States of America; 6 Department of Biostatistics, The University of Texas MD Anderson Cancer Center, Houston, Texas, United States of America; University of Florida, United States of America

## Abstract

**Purpose:**

Folate metabolism, with its importance to DNA repair, provides a promising region for genetic investigation of lung cancer risk. This project investigates genes (*MTHFR*, *MTR*, *MTRR*, *CBS*, *SHMT1*, *TYMS*), folate metabolism related nutrients (B vitamins, methionine, choline, and betaine) and their gene-nutrient interactions.

**Methods:**

We analyzed 115 tag single nucleotide polymorphisms (SNPs) and 15 nutrients from 1239 and 1692 non-Hispanic white, histologically-confirmed lung cancer cases and controls, respectively, using stochastic search variable selection (a Bayesian model averaging approach). Analyses were stratified by current, former, and never smoking status.

**Results:**

Rs6893114 in *MTRR* (odds ratio [OR] = 2.10; 95% credible interval [CI]: 1.20–3.48) and alcohol (drinkers vs. non-drinkers, OR = 0.48; 95% CI: 0.26–0.84) were associated with lung cancer risk in current smokers. Rs13170530 in *MTRR* (OR = 1.70; 95% CI: 1.10–2.87) and two SNP*nutrient interactions [betaine*rs2658161 (OR = 0.42; 95% CI: 0.19–0.88) and betaine*rs16948305 (OR = 0.54; 95% CI: 0.30–0.91)] were associated with lung cancer risk in former smokers. SNPs in *MTRR* (rs13162612; OR = 0.25; 95% CI: 0.11–0.58; rs10512948; OR = 0.61; 95% CI: 0.41–0.90; rs2924471; OR = 3.31; 95% CI: 1.66–6.59), and *MTHFR* (rs9651118; OR = 0.63; 95% CI: 0.43–0.95) and three SNP*nutrient interactions (choline*rs10475407; OR = 1.62; 95% CI: 1.11–2.42; choline*rs11134290; OR = 0.51; 95% CI: 0.27–0.92; and riboflavin*rs8767412; OR = 0.40; 95% CI: 0.15–0.95) were associated with lung cancer risk in never smokers.

**Conclusions:**

This study identified possible nutrient and genetic factors related to folate metabolism associated with lung cancer risk, which could potentially lead to nutritional interventions tailored by smoking status to reduce lung cancer risk.

## Introduction

Lung cancer accounted for 15% of all cancer diagnoses in 2010 and 28% of all cancer deaths [1]. Furthermore, the overall 5-year survival rate remains at 15% for all stages of lung cancer combined [1]. While smoking cigarettes is the dominant risk factor for lung cancer, only a fraction of smokers ever develop the disease [2], suggesting that lung cancer development depends on other factors, most likely genetic and other environmental factors (e.g., diet) [2], [3], [4]. Research suggests that dietary folate status [5], [6] and variation within genes that comprise the folate metabolic pathway [7], [8], [9], [10], [11] may be associated with lung cancer risk.

Folate has been shown to be an important nutrient for DNA synthesis, repair, and methylation [8] and therefore, may influence cancer risk. Studies have shown that low folate intake is associated with increased DNA strand breaks, decreased DNA methylation, and reduced DNA repair capacity [12]. High dietary folate intake (defined as above the median control intake level) is associated with a 40% reduction in lung cancer risk among former smokers, after adjusting for age, sex, ethnicity, total energy intake, body mass index, family history of smoking, pack years smoked, and alcohol consumption [13]. The protective effect of increased folate intake also appears to hold among current smokers [14]. More recently, high blood serum levels of vitamin B_6_ and methionine have been found to offer different levels of protection against lung cancer for never, former and current smokers [6].

Aside from dietary folate, genes in the folate metabolic pathway have also been associated with lung cancer risk. Folate genes implicated in lung cancer risk include methylenetetrahydrofolate reductase (*MTHFR*) [7]; thymidylate synthase (*TYMS*) [10], [15]; serine hydroxymethyltransferase 1 (*SHMT1*) [11]; and cystathionine β-synthase (*CBS*) [8]. Other suspected genes include methionine synthase (*MTR*) and methionine synthase reductase (*MTRR*); however, results for these genes have been equivocal [7].

This study investigates the roles of folate status, nutrition, genes and gene-nutrient interactions in the folate metabolic pathway in lung cancer risk. Previous assessments of the association between folate status and lung cancer have focused on nutrition, without consideration of genetic polymorphisms, and studies that have assessed genes in the folate metabolic pathway included only small panels of single nucleotide polymorphisms (SNPs). The current study examines multiple SNPs in important folate genes, while also investigating dietary intake of B vitamins, folate, methionine, betaine, and choline, allowing for the joint assessment of the effects of diet and genetic status and pairwise gene-nutrient interactions on lung cancer risk.

This study uses a more powerful approach than standard methods for detecting genes and nutrients associated with lung cancer, known as stochastic search variable selection (SSVS) [16], [17]. SSVS, a form of Bayesian model averaging, randomly searches through all possible models, guided by the data, to identify the most likely risk factors accounting for the uncertainty of variable selection [17]. We employed stochastic search here for multiple reasons. First, such stochastic search methods have been effective in analyzing SNP data, particularly in genetic association studies (e.g. [17], [18], [19], [20], [21]). Second, simulation studies using the case-control design have demonstrated that SSVS has a greater accuracy to recover the “true model” than standard variable selection methods, such as forward, backward or stepwise selection [17]. Third, other researchers have shown that SSVS outperforms penalized sparse regression [22] and standard lasso techniques [23], especially in problems investigating many SNPs where then number of SNPs and interactions is larger than the sample size [23], [24], [25].

## Methods

### Ethics Statement

This study was approved by both the MD Anderson and The University of Texas Health Science Center at Houston Institutional Review Boards (IRB). The University of Texas Health Science Center at Houston IRB is also the governing IRB for the UT School of Public Health, as a member school of the University of Texas Health Science Center at Houston. This study involved secondary analysis of de-identified data. The original data collection was consented by written informed consent that discussed such analyses.

### Study Population

The study population consisted of histologically confirmed lung cancer cases (n = 1239) and controls (n = 1692) diagnosed between 1995 and 2007 from an ongoing lung cancer case-control study conducted at The University of Texas MD Anderson Cancer Center. Details of the recruitment for the parent study have been published elsewhere [26], [27]. Briefly, newly diagnosed cases of lung cancer were recruited from patients at MD Anderson Cancer Center. Controls (individuals without a previous diagnosis of cancer, except non-melanoma skin cancer) were recruited from Kelsey-Seybold clinics, the largest private physician group in Houston, Texas. The overall recruitment rate was about 75%.

### Dietary and Demographic Data

Dietary information, demographic factors, and smoking history were obtained through a personal interview. Trained interviewers administered a food frequency questionnaire (FFQ) that is a modified version of the National Cancer Institute's Health Habits and History Questionnaire [28]. The FFQ solicited usual intake for the year prior to the interview and included an open-ended food section and behavior-related dietary questions regarding restaurant dining and food preparation. The validity of the Block FFQ has been described across various populations [29], [30], [31]. The questionnaire was modified for the parent study to include ethnic foods commonly consumed in the greater metropolitan area of Houston, Texas. The estimated intake of several nutrients and beverages in controls was comparable to that reported by adults who participated in the National Health and Nutrition Examination Survey (NHANES), 1999–2000 [32], [33], [34].

In the current study, we limit our analysis to non-Hispanic whites to ensure a large enough sample size to stratify by smoking status and minimize confounding due to population stratification. We also focus on this population to keep our inference consistent with that of the earlier studied Spitz model for non-genetic risk of lung cancer [35].

Questions with multiple foods on the FFQ were weighted for each individual food item by the consumption of that food in the NHANES population (as in [36]). Then, the nutrient content of each food was derived from the U.S. Department of Agriculture National Nutrient Database for Standard Reference, Release 21 (USDA SR21) [37]. Thus, weighted averages of the individual foods in multiple food items as well as the items with one food were linked to the USDA SR21 to calculate nutrient intakes. We determined the daily nutrient intake of the following macro- and micronutrients: energy, carbohydrate, fat, protein, betaine, choline, methionine, folate, pantothenic acid (vitamin B_5_), niacin (vitamin B_3_), riboflavin (vitamin B_2_), thiamin (vitamin B_1_), vitamin B_6_, and vitamin B_12_. All nutrient values were adjusted for total energy intake per the method of Willett and Stampher [38].

In our analysis, alcohol consumption reported on the FFQ was first dichotomized into non-drinkers (reported 0 drinks on the FFQ) versus drinkers (reported any drinking). After significance was assessed, we later categorized alcohol into: nondrinker, 0.1–4.9 g/day, 5.0–14.9 g/day, 15–29.9 g/day, and greater than 30 g/day as in [39] for comparative discussion purposes. We categorized smoking status as never (smoked fewer than 100 cigarettes in their lifetime), former (smoked at least 100 cigarettes in their lifetime and quit more than 1 year prior to study enrollment), or current (currently smoking or quit less than 1 year prior to enrollment) smokers [35]. Family history of smoking-related cancer in a first-degree relative was included in the analysis on the basis of a yes/no response. For all smokers, we computed pack years. For former smokers, we additionally computed years since cessation. For never and former smokers, we recorded exposure to environmental tobacco smoke, defined as exposure to someone else's cigarette smoke at work or at home on a regular basis, as described in [35].

### SNP Selection and Genotyping

We selected 293 SNPs across the genes in the folate metabolism pathway. The full panel of SNPs genotyped, their function and location are given in Table S1. These SNPs consist of all those listed in the HapMap and National Institute of Environmental Health Sciences (NIEHS) SNP databases as members of the above- mentioned genes of the folate metabolism pathway. We consider a SNP to belong to a gene if it is located within 500 kilo base pairs (kb) of the gene. No other genes with known function in folate metabolism have been implicated in lung cancer risk, so we focus our analyses on this set. Thus, our custom chip was composed of SNPs from these genes in the folate pathway. The selected SNPs were genotyped using the custom iSelect Infinium Beadchip design in conjunction with SNPs for other projects.

Participants' genomic DNA was extracted from peripheral blood lymphocytes and stored at −80°C until use. We genotyped SNPs from case and control DNA samples using Illumina's BeadXpress platform according to the standard 3 day protocol. Genotypes were autocalled using the BeadStudio software. SNPs with genotype call rates of less than 95% or SNPs with a minor allele frequency less than 0.01, or more than 10% missing across our data set were omitted from our analysis (39 SNPs removed). A chi-square test confirmed that the pattern of missing observations for each SNP was independent of the affection status of the subjects. For the multivariable analysis, individuals missing SNPs were removed, and 2,225 subjects (1175 cases, 1050 controls) remained in the analysis.

Once the data set was reduced to genotypes not missing SNPs, we reduced the dimensionality and collinearity by empirically selecting tag SNPs using the method of Carlson et al. [40], with a threshold of r^2^ = 0.8. We selected representative tag SNPs that were in exons, or previously mentioned in prior studies, when available. We examined Hardy-Weinberg proportions for the tag SNPs using PLINK [41], and all tag SNPs were found to be in Hardy-Weinberg equilibrium at the 0.001 level.

### Statistical Analysis

Continuous demographic variables were compared using two-sample t-tests; nutrient variables were compared using Wilcoxon rank-sum tests; and categorical demographic variables were compared with Pearson's chi-squared test. For model selection, we used a Bayesian model averaging method known as stochastic search variable selection (SSVS) [16] applied to logistic regression [17], [20]. SSVS uses Markov Chain Monte Carlo (MCMC) methods to search through all possible models to identify joint genetic and dietary effects on lung cancer risk. These methods have been shown to be more powerful than traditional stepwise selection methods [17], [20]. SSVS has two levels of prior distributions: the prior on the model coefficient or odds ratio, which includes a correlation matrix for genetic factors defined by linkage disequilibrium (LD), and the prior for probability of selection for each variable [17], [20].

#### Prior Distribution

To conform with fully Bayesian methods, we modeled the prior correlation among the tag SNPs to be analyzed using the pairwise r^2^ values from the NIEHS Environmental Genome Project [42], external to our data. SNPs more than 400,000 base pairs apart or from different chromosomes were assumed to be independent. As a reliable estimate for the correlation of nutrient values could not be externally defined, the priors for the dietary coefficients were independent normal distributions centered at 0 [17]. We assumed gene-environment independence and modeled the priors for the coefficients for the genes and nutritional covariates as uncorrelated. We also used a prior probability of inclusion of 0.5 for each variable, which has been shown to best control for both false-positive and false-negative results when the prior information for all risk factors may not be available [17].

#### Smoking Variables and Additional Covariates

Because smoking is a well-established risk factor for lung cancer, cases and controls were frequency matched by smoking status in the original study design. Thus, we stratified subjects into three groups based on smoking history: never smokers, former smokers, and current smokers as in Spitz et al. [35]. Other non-genetic risk factors for lung cancer that have been established as significant were included in each model following the approach in Spitz et al. [35], and were not subject to variable selection. For never smokers, we included sex, age, family history of cancer, and exposure to environmental tobacco smoke. For former smokers, we included sex, age, family history of cancer, and a factor indicating whether the subject stopped smoking before age 40, between ages 40 and 53, or at age 54 or later, selected for its stronger association with lung cancer risk than pack years smoked (as described in [35]). For current smokers, we included sex, age, family history of cancer, and a factor indicating whether pack years smoked were less than 27, between 27 and 53, between 54 and 82, or 83 or greater, as in [35]. Genotypes were coded additively, using homozygous for the major allele as the reference genotype.

#### Markov Chain Monte Carlo Analysis

All MCMC computations were completed using WinBugs [43], R and the R2WinBUGS package [44] to prepare the data (categorization, clean missing, stratification) and to compute posterior inference. We ran two chains with distinct starting values for 300,000 iterations and used the last two thirds of the iterations to estimate our posterior quantities to ensure convergence to the stationary distribution, as described in [45]. The two chains for each stratum were found to have very high correlations, indicating that they converged to the same model, and were pooled for inference.

**Figure 1:**
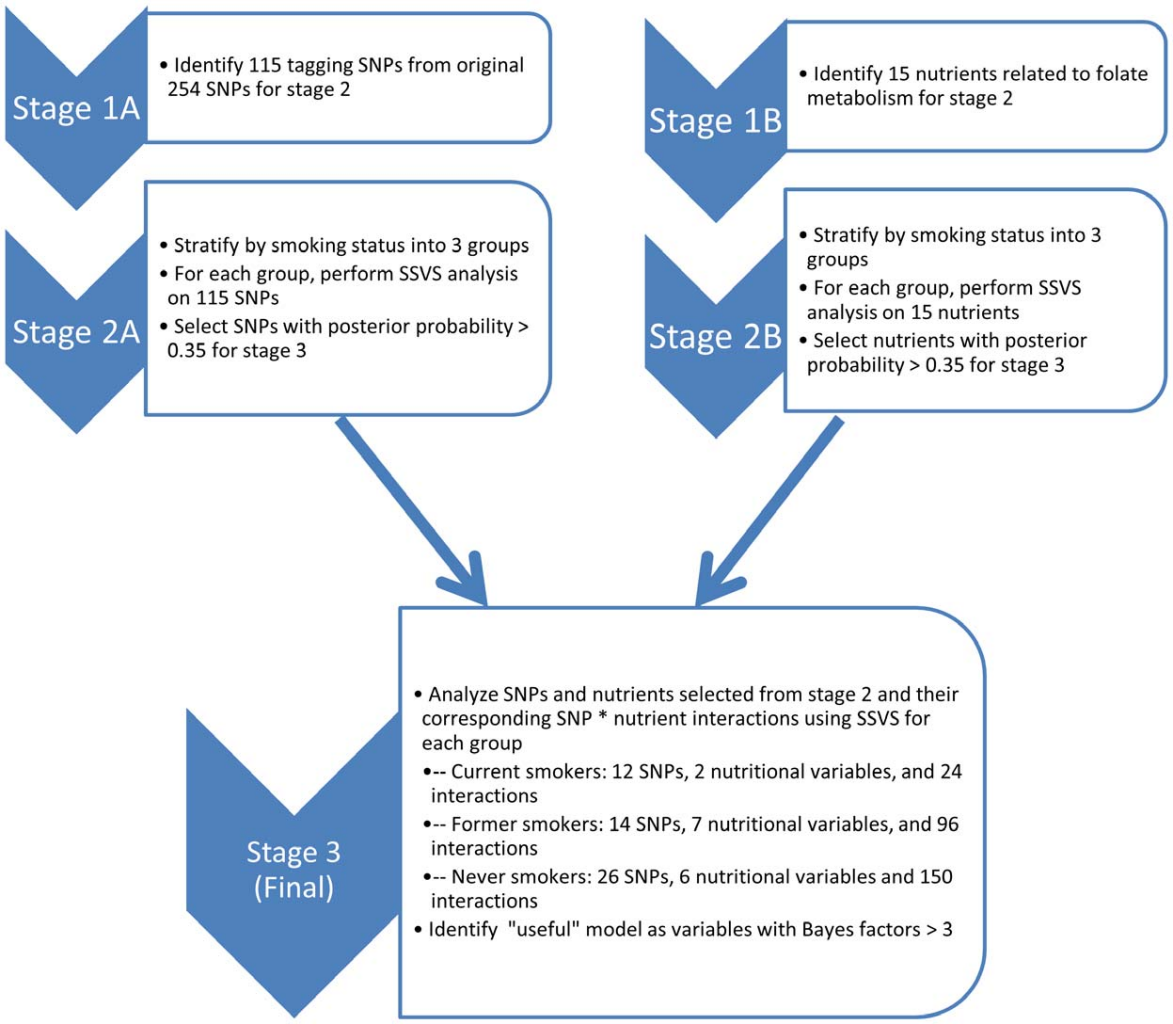
Analysis Flow Chart. This figure depicts the flow of analysis. We analyzed SNPs and nutrients in parallel, using stochastic search methodology in stage 2. Then the most important SNPs and nutrients were jointly investigated along with the gene-nutrient interactions in stage 3, again using stochastic search methodology.

The statistical analysis proceeded in three stages (see Figure 1): In stage 1, we identified the tag SNPs and nutrients to be analyzed. Stage 2 consisted of a stochastic search of the SNPs and a separate stochastic search of the nutrients to screen for promising SNPs and nutrients. Any SNPs and nutrients with a posterior probability of inclusion greater than 0.35 proceeded to stage 3 [17], [46]. In stage 3, we jointly searched through SNPs and nutrients and their corresponding SNP and nutrient interactions for those SNPs and nutrients that proceeded to stage 3. The gene-nutrient interactions consisted of the pairwise product of each SNP additively coded and the nutrient as a continuous variable. For the stage 3 model, we selected genes, nutrients and interactions with a marginal Bayes factor greater than 3 which indicates moderate evidence for association [47] (marginal Bayes factors were computed similar to the SNP specific Bayes factor in [48], based on the marginal probabilities of inclusion). In addition to computing the Bayes factor, we also computed the expected false discovery rate, as defined in [49]. The Bayes factor of 3 or greater also coincides with controlling the false discovery rate to less than 0.15. We estimated the odds ratios (ORs) using the posterior model averaged coefficients, conditional on inclusion and their 95% credible intervals (95% CI), as in [20].

#### Sample Size for SNP Analysis

There were 763 current smokers with complete genotype and covariate data: 406 cases and 357 controls. There were 719 former smokers: 453 cases and 266 controls, and 743 never smokers: 316 cases and 427 controls.

#### Sample Size for Nutritional Analysis

There were 545 current smokers with full nutritional and covariate data available: 250 cases and 295 controls. There were 547 former smokers: 319 cases and 228 controls, and there were 685 never smokers: 279 cases and 406 controls.

#### Determining the final model

The following sample sizes reflect the numbers of subjects with available genotype and nutritional data. Of the current smokers, there were 577 subjects, with 263 cases and 314 controls, in whom we investigated 12 SNPs and 2 nutritional variables and 24 interaction terms. Of the former smokers, there were 572 subjects, with 337 cases and 235 controls, in whom we investigated 14 SNPs and 7 nutritional variables and 96 interactions. For never smokers, the sample size was 743, with 304 cases and 439 controls, and we investigated 26 SNPs and 6 nutritional variables and 150 interactions. Highly collinear interactions were dropped from the selection process (2 interactions for former smokers, and 6 interaction terms for never smokers).

## Results

We summarize selected demographic variables and nutritional variables in our study population in Table 1. There were equal proportions of male and female cases (50.5% versus 49.5%, respectively) but slightly fewer male (46.7%) than female controls (53.3%). The mean age of cases was significantly higher than the mean age of controls (63.5 years versus 57.2 years, p<0.001). Our sample had a higher proportion of former and current smokers in the cases (72.4%) than in the controls (58.8%). Cases who smoked had significantly more pack years than controls (mean 78.3 pack years versus mean 59.0 pack years, p<0.001). (Our analyses were stratified by smoking status and adjusted for pack years.) More controls (41.1%) were never smokers than cases (27.5%). Current and former smokers reported exposure to environmental tobacco smoke in close to equal proportions of cases and controls (68.2% versus 68.5%, respectively), and significantly more cases than controls reported having at least one relative with a smoking-related cancer (30.3% versus 21.2%, respectively).

**Table 1:** Distribution of Epidemiologic/Demographic Variables and Nutrition Variables.

Variable^a^	Category	Cases (N = 1692)	Controls (N = 1239)	P-value^b^
**Gender, n (%)**	Male	854 (50.5)	579 (46.7)	
	Female	838 (49.5)	660 (53.3)	0.049
**Age, mean (SD)**		63.5 (11.0)	57.2 (13.2)	<0.001
**Smoking status, n (%)**	Never	466 (27.5)	510 (41.1)	
	Former	645 (38.1)	326 (26.3)	
	Current	581 (34.3)	403 (32.5)	<0.001
**Pack years** c **, mean (SD)**		78.3 (73.7)	59.0 (49.8)	<0.001
**ETS** d **, n (%)**	Exposed	236 (68.2)	322 (68.5)	
	Unexposed	110 (31.8)	148 (31.5)	0.97
**Family history, n (%)**	None	936 (69.7)	938 (78.8)	
	≥1 relative	406 (30.3)	253 (21.2)	<0.001
**Nutrition, median**	Total calories (kcal)	1734.91	1717.92	0.752
	Alcohol (g)	0.27	0.82	0.002
**Macronutrients** e	Carbohydrate (g)	233.95	231.80	0.256
	Protein (g)	73.58	76.43	<0.001
	Total Fat (g)	67.82	67.98	0.876
**Micronutrients** e	Betaine (mg)	48.94	53.20	0.003
	Choline (mg)	141.15	143.04	0.316
	Folate (mg)	501.19	514.48	0.344
	Methionine (mg)	1489.22	1554.95	<0.001
	Niacin (mg)	21.99	22.67	0.005
	Pantothenic acid (mg)	5.43	5.33	0.116
	Riboflavin (mg)	2.23	2.23	0.661
	Thiamin (mg)	1.47	1.47	0.801
	Vitamin B_6_ (mg)	2.00	2.07	0.012
	Vitamin B_12_ (mg)	5.26	5.42	0.005

a Totals may not equal total N due to missing data.

b P-value from the two-sided chi-squared test (for categorical variables), Student's t-test (for continuous demographic variables), or Wilcoxon rank sum test (for nutrition variables).

c Pack years computed for former and current smokers only.

d ETS = Environmental Tobacco Smoke exposure in never smokers.

e Macro- and micronutrients are adjusted by total calorie intake.

Only a few dietary factors differed significantly across cases and controls. Considering the median grams of alcohol drunk, cases reported significantly less drinking (median = 0.27 g) than controls (median = 0.82 g). Cases reported eating significantly less protein (median = 73.58 g) than controls (76.43 g). Cases also reported significantly less betaine (median = 48.94 mg), methionine (median = 1480.22 mg), niacin (median = 21.99 mg), vitamin B_6_ (median = 5.43 mg), and vitamin B_12_ (median = 5.26 mcg) than controls (medians = 53.20 mg, 1554.95 mg, 22.67 mg, 2.07 mg, 5.42 mcg, respectively).

### Initial Screen for Genotypes

Our initial screen for SNPs associated with lung cancer stratified by smoking status is reported in Table S2. Those SNPs with a PPI greater than 35% were further analyzed in the final model that jointly considered genes and nutrition. Among current smokers, we identified 12 SNPs for further consideration: 2 SNPs from the *CBS* gene, 7 SNPs from *MTRR*, 2 SNPs from *SHMT1*, and 1 SNP from *TYMS*. In former smokers, we identified 14 SNPs: 3 SNPs from the *CBS* gene, 2 from *MTHFR*, 1 from *MTR*, 7 from *MTRR*, and 1 from *TYMS*. In never smokers, we identified 26 SNPs: 4 in *CBS*, 8 in *MTHFR*, 11 in *MTRR*, 1 in *SHMT1*, and 2 in *TYMS*.

### Initial Screen for Nutrients

The results from our initial screen for potential nutrients associated with lung cancer are reported in Table S3, stratified by smoking status. Those nutrients with a PPI greater than 35% were further analyzed in our final model that jointly considered genes and nutrition. In current smokers, only alcohol and vitamin B_6_ were identified for consideration in the final model. In former smokers, we identified alcohol, carbohydrates, protein, betaine, methionine, thiamin, and vitamin B_12_ for further consideration. In never smokers, the nutrients for further consideration were carbohydrates, protein, choline, folate, riboflavin, and thiamin.

### Final Models

The collinearity of variables in our final model was controlled using the tag SNP selection process described above. We include the LD matrix describing the LD between the final selected SNPs in Table S4 (max r^2^<0.63) and the correlations between the final selected nutrients in Table S5 (max r^2^<0.62). Simulation studies have shown stochastic search to perform well in the presence of moderate collinearity of magnitude on the order of 0.6 [20].

#### Current Smokers

In current smokers, *MTRR* (rs6893114) and alcohol were associated with lung cancer risk, after adjusting for sex, age, pack years, and family history (Table 2). *MTRR* had the highest PPI, and the minor allele of rs6893114 conferred a twofold increase in lung cancer risk (OR = 2.10; 95% CI: 1.20–3.48). As alcohol drinking appeared to be protective among current smokers (OR = 0.48; 95% CI: 0.26–0.84), we further examined alcohol intake using more detailed categorization [39]: nondrinker, 0.1–4.9 g/day, 5.0–14.9 g/day, 15–29.9 g/day, and greater than 30 g/day. We computed an adjusted odds ratio for each level (see Table 3), using non-drinkers as the reference category and adjusting for age, sex, family history, pack years, and the *MTRR* variant. The two lowest drinking categories (light and moderate drinking) showed a decrease in risk from drinking: 0.1–4.9 g/day is associated with a 39% decrease in risk, (OR = 0.61; 95% CI: 0.40–0.93) and 5–14.9 g/day is associated with a 42% decrease in risk (OR = 0.58; 95% CI: 0.34–0.99). We did not find any evidence of gene-nutrient interactions for current smokers in our data.

**Table 2:** Final Model Stratified by Smoking Status.

Model	Variable	Gene^a^	OR^b^	95% CI	PPI	Bayes factor
**Current Smokers**	alcohol		0.48	0.26–0.84	0.90	8.81^c^
	rs6893114	*MTRR*	2.10	1.20–3.48	0.88	7.25^c^
**Former Smokers**	betaine*rs2658161	*MTRR*	0.42	0.19–0.88	0.82	4.62^c^
	rs13170530	*MTRR*	1.70	1.10–2.87	0.81	4.21^c^
	betaine*rs16948305	*TYMS*	0.54	0.30–0.91	0.76	3.10^c^
	rs2658161	*MTRR*	2.23	0.96–4.98	0.72	2.22
	betaine		0.71	0.34–1.35	0.32	0.47
	rs16948305	*TYMS*	0.89	0.54–1.62	0.23	0.30
**Never Smokers**	rs2924471	*MTRR*	3.31	1.66–6.59	0.99	78.31^c^
	rs13162612	*MTRR*	0.25	0.11–0.58	0.98	65.63^c^
	rs10512948	*MTRR*	0.61	0.41–0.90	0.83	5.17^c^
	choline*rs10475407	*MTRR*	1.62	1.11–2.42	0.82	4.83^c^
	riboflavin*rs876712	*MTRR*	0.40	0.15–0.95	0.80	3.98^c^
	choline*rs11134290	*MTRR*	0.51	0.27–0.92	0.78	3.48^c^
	rs9651118	*MTHFR*	0.63	0.43–0.95	0.75	3.05^c^
	rs11134920	*MTRR*	0.68	0.44–1.05	0.48	0.92
	riboflavin		1.57	0.65–4.03	0.45	0.81
	rs10475407	*MTRR*	0.77	0.57–1.04	0.38	0.62
	choline		0.75	0.35–1.78	0.36	0.55
	rs876712	*MTRR*	0.73	0.41–1.29	0.35	0.55

a SNPs located within 500 kb of given gene.

b Odds ratios for current smokers adjusted for sex, age, family history of smoking-related cancers, and pack years smoked; odds ratios for former smokers adjusted for sex, age, family history, and age at smoking cessation; odds ratios for never smokers adjusted for sex, age, family history, and exposure to secondhand tobacco smoke.

c Bayes factor greater than or equal to 3; included in the final model.

**Table 3:** Further Examination of Alcohol from the Final Model for Current Smokers (263 Cases/314 Controls).

Alcohol Intake Category	Cases	Controls	OR^a^	95% CI
Non-drinkers	116	86	1	Reference
0.1–4.9 g/day	72	111	0.61	0.40–0.93
5–14.9 g/day	31	58	0.58	0.34–0.99
15–29.9 g/day	21	30	0.73	0.38–1.37
>30 g/day	23	29	0.75	0.40–1.40

a Odds ratios adjusted for age, sex, family history, pack years, and *MTRR* mutants.

#### Former Smokers

For former smokers, one SNP in *MTRR* (rs13170530) showed evidence of an association with lung cancer risk after adjusting for age, sex, age at smoking cessation, and family history. We also found a significant interaction between betaine and a variant in *MTRR* (rs2658161) and a variant in *TYMS* (rs16948305). The minor allele in *MTRR* (rs13170530) confers a 70% increase in risk (OR = 1.70; 95% CI: 1.10–2.87). Although no nutrient main effects were significant, betaine was a part of two meaningful interactions that were both protective among former smokers: betaine*rs2658161 (OR = 0.42; 95% CI: 0.19–0.88) and betaine*rs169484305 (OR = 0.54, 95% CI: 0.30–0.91). Thus each copy of the minor allele of rs2658161 improves the protective effect of ingesting more betaine. Zero copies of the minor allele results in no reduction in risk, 1 allele results in a 58% reduction in risk per mg increase in betaine intake, while 2 copies of the minor allele result in 0.83% reduction in risk. Individuals heterozygous for the minor allele of rs169484305 receive a 46% decrease in lung cancer risk per mg betaine consumed, while those homozygous for the minor allele have a 70% decrease in risk.

#### Never Smokers

For never smokers, 4 SNPs, three in *MTRR* (rs13162612, rs10512948, rs2924471) and one in *MTHFR* (rs9651118), were associated with lung cancer risk, after adjusting for environmental tobacco exposure, sex, age, and family history. The minor allele for rs13162612 was associated with a 75% reduction in lung cancer risk (OR = 0.25; 95% CI: 0.11–0.58), and rs10512948 was associated with a 39% decrease in lung cancer risk (OR = 0.61; 95% CI: 0.41–0.90). The third SNP from *MTRR*, rs2924471, was associated with a 3-fold increased risk (OR = 3.31; 95% CI: 1.66–6.59). In addition to the genetic main effects, three nutrient by gene interactions were selected: choline*rs10475407, choline*rs11134290 and riboflavin*rs876712. The choline*rs10475407 interaction conferred risk (OR = 1.62; 95% CI: 1.11–2.41). Being heterozygous for the minor allele of rs10475407 is associated with a 62% increased risk, while being homozygous for the minor allele is associated with a 2.6 fold increase in risk for each mg increase of choline intake. On the other hand, individuals heterozygous for the minor allele of rs11134290 had a 49% decrease in risk (OR = 0.51; 95% CI: 0.27–0.92) for increased choline intake, while those homozygous for the minor allele had a 74% decreased risk per mg increased intake in choline. Individuals heterozygous for the minor allele of rs876712 have a 60% decreased risk per increased intake in riboflavin, which increases to 84% for those homozygous for the minor allele (riboflavin*rs876712 OR = 0.40; 95% CI: 0.15–0.95).

## Discussion

This study identified various nutritional factors and genetic factors related to folate metabolism that jointly play a role in lung cancer risk. Performing analyses stratified by smoking status (current, former, and never), we found folate-related dietary and genetic factors, and gene*nutrient interactions were associated with lung cancer risk in current, former, and never smokers. Alcohol was associated with lung cancer risk in current smokers, while gene-nutrient interactions were associated with varying risk in former and never smokers. SNPs in *MTRR* were associated with lung cancer risk in current, former and never smokers, while a variant in *MTHFR* was associated with lung cancer risk in never smokers. An additional SNP in *TYMS* was found to interact with betaine to influence lung cancer risk in former smokers.

Recent research has shown mixed results regarding the association between alcohol drinking and lung cancer risk in non-Hispanic whites [39], [50], [51], [52]. Evidence is more consistent in never smokers for no association between alcohol and lung cancer risk [50], which aligns with the findings of the current study. For current smokers, there is less evidence regarding the association between alcohol intake and lung cancer risk [51]. Recent studies suggest that the influence of alcohol may depend on the type of alcohol consumed, citing a possible protective effect for wine and increased risk for beer [52]. Other studies show a marginal, non-linear relationship between alcohol intake and lung cancer risk, with moderate drinking having a protective effect [39]. The strong posterior probability for alcohol seen here suggests that alcohol may be associated with lung cancer risk in current smokers; however, this study does not provide any definitive resolution regarding the mediating effects of smoking on the relationship between alcohol and lung cancer risk. Possible explanations for this apparent association are that cases stopped drinking recently relative to their diagnosis or simply under-report their drinking because of recent diagnosis.

Our analysis also identified several polymorphisms associated with lung cancer risk. For all smoking statuses, different SNPs in *MTRR* exhibited strong evidence for association with lung cancer risk. In current smokers, we identified rs6893114 with increased risk, for former smokers, we identified rs13170530 with increased risk, and for never smokers we identified multiple *MTRR* SNPs: rs13162612 and rs10512948 were associated with decreased risk; and rs2924471 was associated with increased risk for lung cancer. There is very little information regarding the association of *MTRR* with lung cancer, with most studies focusing on *MTRR A66G* (rs1801394) [7], [9], [53]. Two previous studies found no association for *MTRR A66G* [7], [53], while a third found increased risk [9]. All three studies mentioned an interaction between smoking status and *A66G* alleles. The fact that the current study also found polymorphisms in *MTRR* provides further evidence of an association between *MTRR* and lung cancer.

In never smokers, an additional polymorphism in *MTHFR*, rs9651118, is associated with decreased lung cancer risk. Over the past decade, many researchers have focused their efforts on two particular polymorphisms of *MTHFR*, *C677T* and *A1289C*, owing to variants from wild-type at these loci resulting in altered serum folate levels [7], [54], [55]. However, results concerning these two loci and their association with lung cancer risk are often inconclusive [54], [56]. In the current study, *C677T* was not selected as being associated with lung cancer. The SNP associated with lung cancer in never smokers, rs9651118 (T/C), has a borderline Bayes factor (3.05), and moderate protective effect for lung cancer (37% decrease in risk). This SNP is in low LD with the *C677T* and *A1298C* polymorphisms for *MTHFR* (r^2^<0.20) and is located in an intronic region of *MTHFR*. (We do not use D′ here, because by D′, all tag SNPs have D′>0.9 with *C677T*.) Given the findings in this study, further investigation of this SNP is encouraged.

Located at 1p36.3, the *MTHFR* gene codes for the methyenetetrahydrofolate reductase that converts 5,10-methylenetetrahydrofolate to 5-methylenetetrahydrofolate, which is the primary circulating form of folate and provides methyl groups for synthesis of methionine, an important factor for healthy DNA methylation. *MTRR* codes for methionine synthase reductase, which controls methionine synthase, which uses methionine as a methyl donor for DNA methylation. A disruption in any of these three metabolites can lead to chromosome instability and DNA under-methylation, and ultimately to cancer [57], [58]. *TYMS* codes for thymidylate synthase, an enzyme that is key to a reaction providing thymidine, an important nucleotide used in DNA synthesis and repair. Increased activity is expected to be associated with healthier DNA, while decreased activity is expected to be associated with more DNA damage and thus higher cancer risk [10], [53].

Our analysis did not detect any nutrient main effects; however, for each smoking status we did detect statistical interactions. In former smokers, we detected a statistical interaction between variants in *MTRR* and *TYMS* and betaine, and in never smokers we detected interactions between variants in *MTRR* and choline and riboflavin. The Bayes factors indicated no evidence for any associations between lung cancer and the main effects of betaine, choline or riboflavin or the SNPs involved in the interactions, but as the intake of betaine and allele dose of rs2658161 (*MTRR*) and rs16948305 (*TYMS*) increased, our model indicated a reduction in lung cancer risk for former smokers. With never smokers, a statistical interaction with choline and rs10475407 (*MTRR*) lead to an increased risk, while the interactions of choline with rs11134290 (*MTRR*) and riboflavin with rs876712 (*MTRR*) were modeled to decrease lung cancer risk. Researchers are just beginning to investigate choline and betaine intake in human studies, due to the recently available database linkage to FFQs for betaine and choline [59]. Some human studies have linked breast cancer [60] and colon cancer [61] to choline and betaine intake levels, while other studies have found no association [62], [63], [64]. Riboflavin has been reported with mixed associations with lung cancer as well [6], [65], [66]. Therefore, the literature offers other studies that support many of the SNPs found by this Bayesian model averaging method. However, this is one of the first studies to jointly model these risk factors for lung cancer, and further validation of these findings is needed.

The findings of the current study need to be interpreted in the light of certain limitations especially for the nutrition data. First, because this study sample was restricted to non-Hispanic whites, our findings may not generalize to other ethnicities. Second, this study is a cross-sectional study and information on all variables was collected upon recruitment, and we cannot investigate any real change in behavior over time, such as a change in drinking behavior. Third, the controls were selected from an HMO in the greater Houston metropolitan area. Therefore, controls may not be fully representative of the general population. The fact that these individuals sought medical care might suggest a higher awareness of health and, perhaps, of the importance of proper nutrition. Therefore, the nutrition profiles may not accurately reflect intake in the general population. However, a previous study found the intake of various food items in this population to be comparable to those found by NHANES [32], [33], [34].

Additionally, the pattern of missing data is significantly different in smokers versus non-smokers, but since we stratify by smoking status, the bias will be minimal. The missing pattern between cases and controls are not significantly different. The nutrition data were collected using food frequency questionnaires, which have the well-known limitations of recall bias [67], minimized in this study by interviewer administration. Even though this bias was minimized by administration by trained interviewers, it may be a factor contributing to the difference in the findings here compared to results found using prospective data such as EPIC [6] and ATBC [5]. Once we removed the missing data, and stratified, the sample sizes are small. Yet using the Bayesian approach, we were able to control the false discovery rate to be less than 15%, which for the number of findings of the study, comes to one expected false positive per model. Even though the false discovery rate was controlled, and recall bias was minimized, an important next step is to externally validate these findings with independent, prospectively collected data sets.

We would also like to discuss our independence assumptions. When constructing our priors, we modeled genetic covariance using linkage disequilibrium, but assumed nutrition variables and gene-nutrition interactions to be independent. Prior definitions are not rigid assumptions, but rather reflection of the prior belief of the modeler [68]. Previous simulation studies involving LD as a prior showed that it can reduce false positives from multicollinearity in the presence of high LD [20], [69], [70]. This covariance argument can generalize to correlation between any covariates. As a secondary precaution we computed the false discovery rate as described in [49], and it was controlled at around 15%.

To our knowledge, this is one of the first studies to jointly assess the association between lung cancer and a comprehensive panel of candidate genes in the folate pathway and nutrients related to folate metabolism, and nutrient-gene interactions. Furthermore, we used a novel Bayesian model averaging method to explore these associations. Strengths of this study include a sample size large enough to stratify by smoking status and jointly investigate multiple factors. Jointly modeling gene and nutrient factors allowed us to comprehensively assess the impact of folate metabolism and lung cancer risk. Through our stratified models, we also show that the genetic and nutritional impact on lung cancer risk differs by smoking status. These preliminary findings suggest that the impact of dietary interventions for lung cancer risk may be modified by genotypes in key folate metabolism genes. These findings mark a first step toward more personalized interventions to reduce cancer risk. In developing dietary interventions to reduce lung cancer risk, we not only need to consider smoking status, but also potentially, the genotypes of folate metabolism genes, and how they interact with the nutrient intake levels.

## Supporting Information

Table S1 SNP location and Function. Lists all SNPs analyzed and their minor allele frequency, gene, and function or location by RS number.
(DOCX)

Table S2 SNPs selected from initial screening stratified by smoking status. Lists all snps that passed the first screen of association using PPI greater than 0.35 for each smoking status.
(DOCX)

Table S3 Nutrients Selected from Initial Screening Stratified by Smoking Status. Table listing nutrients that passed the first screen of association using PPI greater than 0.35, for each smoking status.
(DOCX)

Table S4 Linkage Disequilibrium Magnitudes Between SNPs in the Final Model. Table listing the absolute LD magnitudes for thos SNPs identified in any final model.
(DOCX)

Table S5 Correlation Between Nutrients in Final Model. Table listing the correlation between nutrients identified in any final model.
(DOCX)
