# Supplementary material for: Investigating Multiple Candidate Genes and Nutrients in the Folate Metabolism Pathway to Detect Genetic and Nutritional Risk Factors for Lung Cancer
Source: PLoS One. 2013 Jan 23;8(1):e53475. doi: 10.1371/journal.pone.0053475 (PMC3553105; doi:10.1371/journal.pone.0053475)
Supplement: Table S1 — SNP location and Function. Lists all SNPs analyzed and their minor allele frequency, gene, and function or location by RS number. (DOCX) [file pone.0053475.s001.docx]

Supplementary Table S1: SNP Location and Function^a^

| **Reference SNP id** | **Chrom** | **Position** | **Minor allele** | **Minor allele freq** | **Gene**^b^ | **Function or location** |
| --- | --- | --- | --- | --- | --- | --- |
| rs6696752 | 1 | 11835935 | T | 0.26 | *MTHFR* | intron-variant |
| rs4846048 | 1 | 11846252 | G | 0.26 | *MTHFR* | utr-variant-3-prime, intron-variant |
| rs2184226 | 1 | 11847436 | C | 0.05 | *MTHFR* | utr-variant-3-prime, intron-variant |
| rs3737967 | 1 | 11847449 | A | 0.05 | *MTHFR* | missense, utr-variant-3-prime |
| rs1537516 | 1 | 11847861 | A | 0.09 | *MTHFR* | utr-variant-3-prime, intron-variant |
| rs4846049 | 1 | 11850365 | T | 0.31 | *MTHFR* | utr-variant-3-prime |
| rs2274976 | 1 | 11850927 | T | 0.05 | *MTHFR* | missense |
| rs3818762 | 1 | 11851003 | C | 0.22 | *MTHFR* | intron-variant |
| rs13306556 | 1 | 11852110 | T | 0.09 | *MTHFR* | intron-variant |
| rs1476413 | 1 | 11852300 | T | 0.22 | *MTHFR* | intron-variant |
| rs17375901 | 1 | 11852516 | T | 0.03 | *MTHFR* | intron-variant |
| rs12121543 | 1 | 11854671 | A | 0.19 | *MTHFR* | intron-variant |
| rs1994798 | 1 | 11854755 | G | 0.36 | *MTHFR* | intron-variant |
| rs2066462 | 1 | 11854896 | A | 0.09 | *MTHFR* | synonymous-codon |
| rs6541003 | 1 | 11855867 | G | 0.35 | *MTHFR* | intron-variant |
| rs1801133 | 1 | 11856378 | A | 0.32 | *MTHFR* | missense |
| rs17421462 | 1 | 11856847 | A | 0.04 | *MTHFR* | intron-variant |
| rs1572151 | 1 | 11857711 | C | 0.04 | *MTHFR* | intron-variant |
| rs17421511 | 1 | 11857788 | A | 0.11 | *MTHFR* | intron-variant |
| rs4846052 | 1 | 11857951 | T | 0.42 | *MTHFR* | intron-variant |
| rs17421560 | 1 | 11858324 | A | 0.09 | *MTHFR* | intron-variant |
| rs11121832 | 1 | 11860120 | T | 0.20 | *MTHFR* | intron-variant |
| rs2066471 | 1 | 11860458 | T | 0.11 | *MTHFR* | intron-variant |
| rs13306567 | 1 | 11860465 | G | 0.02 | *MTHFR* | intron-variant |
| rs7533315 | 1 | 11860683 | T | 0.20 | *MTHFR* | intron-variant |
| rs17037390 | 1 | 11860843 | A | 0.13 | *MTHFR* | intron-variant |
| rs17037397 | 1 | 11862163 | A | 0.05 | *MTHFR* | intron-variant |
| rs9651118 | 1 | 11862214 | C | 0.21 | *MTHFR* | intron-variant |
| rs17367504 | 1 | 11862778 | G | 0.11 | *MTHFR* | intron-variant |
| rs2066470 | 1 | 11863057 | A | 0.09 | *MTHFR* | synonymous-codon |
| rs7553194 | 1 | 11864149 | A | 0.09 | *MTHFR* | intron-variant |
| rs3753584 | 1 | 11864586 | C | 0.12 | *MTHFR* | intron-variant, upstream-variant-2KB |
| rs3753582 | 1 | 11865542 | C | 0.09 | *MTHFR* | intron-variant, upstream-variant-2KB |
| rs13306561 | 1 | 11865804 | G | 0.14 | *MTHFR* | intron-variant, upstream-variant-2KB |
| rs12354209 | 1 | 236959252 | G | 0.33 | *MTR* | intron-variant |
| rs10495384 | 1 | 236961355 | T | 0.09 | *MTR* | intron-variant |
| rs10733118 | 1 | 236964010 | T | 0.33 | *MTR* | intron-variant |
| rs2185208 | 1 | 236968356 | C | 0.27 | *MTR* | intron-variant |
| rs7526063 | 1 | 236971998 | T | 0.06 | *MTR* | intron-variant |
| rs12759827 | 1 | 236972186 | G | 0.13 | *MTR* | intron-variant |
| rs4659723 | 1 | 236972728 | T | 0.08 | *MTR* | intron-variant |
| rs4659724 | 1 | 236974124 | A | 0.32 | *MTR* | intron-variant |
| rs955516 | 1 | 236980504 | A | 0.35 | *MTR* | intron-variant |
| rs12060264 | 1 | 236988363 | A | 0.35 | *MTR* | intron-variant |
| rs6682427 | 1 | 236993128 | A | 0.07 | *MTR* | intron-variant |
| rs946403 | 1 | 236996117 | C | 0.45 | *MTR* | intron-variant |
| rs11800413 | 1 | 237002877 | G | 0.37 | *MTR* | intron-variant |
| rs2789352 | 1 | 237003997 | A | 0.26 | *MTR* | intron-variant |
| rs12129440 | 1 | 237014635 | A | 0.13 | *MTR* | intron-variant |
| rs2275568 | 1 | 237015628 | T | 0.43 | *MTR* | intron-variant |
| rs3768139 | 1 | 237027668 | G | 0.26 | *MTR* | intron-variant |
| rs3768142 | 1 | 237028564 | G | 0.37 | *MTR* | intron-variant |
| rs10495386 | 1 | 237032808 | A | 0.09 | *MTR* | intron-variant |
| rs1770449 | 1 | 237038161 | C | 0.26 | *MTR* | intron-variant |
| rs4659738 | 1 | 237040858 | G | 0.36 | *MTR* | intron-variant |
| rs4659739 | 1 | 237040913 | T | 0.36 | *MTR* | intron-variant |
| rs10754584 | 1 | 237044933 | G | 0.29 | *MTR* | intron-variant |
| rs10925257 | 1 | 237046160 | G | 0.19 | *MTR* | intron-variant |
| rs1805087 | 1 | 237048500 | G | 0.19 | *MTR* | missense |
| rs2275566 | 1 | 237048562 | G | 0.27 | *MTR* | intron-variant |
| rs10158822 | 1 | 237050176 | G | 0.19 | *MTR* | intron-variant |
| rs1266164 | 1 | 237050951 | T | 0.26 | *MTR* | intron-variant |
| rs10925261 | 1 | 237053186 | T | 0.19 | *MTR* | intron-variant |
| rs3768152 | 1 | 237053530 | A | 0.07 | *MTR* | intron-variant |
| rs2282369 | 1 | 237054799 | A | 0.31 | *MTR* | intron-variant |
| rs10802569 | 1 | 237055305 | G | 0.35 | *MTR* | intron-variant |
| rs1252252 | 1 | 237056002 | T | 0.26 | *MTR* | intron-variant |
| rs2297965 | 1 | 237057608 | G | 0.45 | *MTR* | intron-variant |
| rs3820571 | 1 | 237060433 | G | 0.28 | *MTR* | intron-variant |
| rs12022937 | 1 | 237060850 | C | 0.17 | *MTR* | intron-variant |
| rs11799670 | 1 | 237061097 | G | 0.08 | *MTR* | utr-variant-3-prime |
| rs2853523 | 1 | 237062198 | A | 0.27 | *MTR* | utr-variant-3-prime |
| rs1050993 | 1 | 237062305 | A | 0.26 | *MTR* | utr-variant-3-prime |
| rs10925263 | 1 | 237063748 | C | 0.15 | *MTR* | utr-variant-3-prime |
| rs10754586 | 1 | 237076653 | A | 0.46 | *MTR* | function unknown |
| rs1252109 | 1 | 237111682 | T | 0.25 | *MTR* | function unknown |
| rs6683896 | 1 | 237129335 | T | 0.49 | *MTR* | function unknown |
| rs1801394 | 5 | 7870973 | G | 0.38 | *MTRR* | missense, upstream-variant-2KB |
| rs326120 | 5 | 7874847 | G | 0.19 | *MTRR* | intron-variant |
| rs2303079 | 5 | 7875307 | G | 0.23 | *MTRR* | intron-variant |
| rs7730643 | 5 | 7875963 | G | 0.18 | *MTRR* | intron-variant |
| rs3776467 | 5 | 7876315 | G | 0.45 | *MTRR* | intron-variant |
| rs326122 | 5 | 7876611 | G | 0.19 | *MTRR* | intron-variant |
| rs326124 | 5 | 7877178 | A | 0.19 | *MTRR* | intron-variant |
| rs161869 | 5 | 7877831 | T | 0.47 | *MTRR* | intron-variant |
| rs1532268 | 5 | 7878179 | T | 0.25 | *MTRR* | missense |
| rs2303080 | 5 | 7878424 | A | 0.04 | *MTRR* | missense |
| rs3776465 | 5 | 7878976 | C | 0.25 | *MTRR* | intron-variant |
| rs7703033 | 5 | 7879950 | A | 0.25 | *MTRR* | intron-variant |
| rs6555501 | 5 | 7879983 | C | 0.45 | *MTRR* | intron-variant |
| rs162031 | 5 | 7880287 | T | 0.27 | *MTRR* | intron-variant |
| rs162032 | 5 | 7880634 | A | 0.19 | *MTRR* | intron-variant |
| rs162033 | 5 | 7880835 | T | 0.47 | *MTRR* | intron-variant |
| rs162034 | 5 | 7882979 | T | 0.22 | *MTRR* | intron-variant |
| rs161871 | 5 | 7884389 | G | 0.32 | *MTRR* | intron-variant |
| rs3776463 | 5 | 7885227 | G | 0.08 | *MTRR* | intron-variant |
| rs16879305 | 5 | 7885526 | C | 0.20 | *MTRR* | intron-variant |
| rs162036 | 5 | 7885959 | G | 0.23 | *MTRR* | missense |
| rs162038 | 5 | 7886131 | G | 0.20 | *MTRR* | intron-variant |
| rs3815743 | 5 | 7887109 | G | 0.14 | *MTRR* | intron-variant |
| rs162039 | 5 | 7887456 | T | 0.25 | *MTRR* | intron-variant |
| rs162040 | 5 | 7887478 | C | 0.17 | *MTRR* | intron-variant |
| rs2287779 | 5 | 7889216 | A | 0.08 | *MTRR* | synonymous-codon |
| rs2287780 | 5 | 7889304 | T | 0.08 | *MTRR* | missense |
| rs16879334 | 5 | 7891506 | G | 0.08 | *MTRR* | missense |
| rs162048 | 5 | 7892310 | G | 0.21 | *MTRR* | intron-variant |
| rs2277957 | 5 | 7892644 | T | 0.08 | *MTRR* | intron-variant |
| rs10380 | 5 | 7897191 | T | 0.19 | *MTRR* | missense |
| rs12347 | 5 | 7897283 | A | 0.22 | *MTRR* | synonymous-codon |
| rs1802059 | 5 | 7897319 | A | 0.25 | *MTRR* | synonymous-codon |
| rs10475399 | 5 | 7898094 | G | 0.49 | *MTRR* | intron-variant |
| rs327593 | 5 | 7898991 | G | 0.03 | *MTRR* | intron-variant |
| rs49672 | 5 | 7899253 | A | 0.20 | *MTRR* | intron-variant |
| rs716537 | 5 | 7899419 | T | 0.24 | *MTRR* | intron-variant |
| rs10520874 | 5 | 7899909 | C | 0.20 | *MTRR* | intron-variant |
| rs9332 | 5 | 7900712 | A | 0.22 | *MTRR* | utr-variant-3-prime |
| rs8659 | 5 | 7900833 | A | 0.48 | *MTRR* | utr-variant-3-prime |
| rs10520873 | 5 | 7901230 | C | 0.20 | *MTRR* | utr-variant-3-prime |
| rs7715062 | 5 | 7906907 | T | 0.29 | *MTRR* | function unknown |
| rs13173919 | 5 | 7919212 | C | 0.08 | *MTRR* | function unknown |
| rs179171 | 5 | 7924914 | C | 0.35 | *MTRR* | function unknown |
| rs1532267 | 5 | 7935012 | A | 0.19 | *MTRR* | function unknown |
| rs10512934 | 5 | 7936287 | C | 0.17 | *MTRR* | function unknown |
| rs329852 | 5 | 7943796 | C | 0.17 | *MTRR* | function unknown |
| rs1471621 | 5 | 7946262 | A | 0.37 | *MTRR* | function unknown |
| rs1564592 | 5 | 7950600 | G | 0.37 | *MTRR* | function unknown |
| rs6555507 | 5 | 7965493 | C | 0.30 | *MTRR* | function unknown |
| rs7448905 | 5 | 7970122 | G | 0.29 | *MTRR* | function unknown |
| rs2170501 | 5 | 7970222 | A | 0.29 | *MTRR* | function unknown |
| rs7728958 | 5 | 7972269 | A | 0.30 | *MTRR* | function unknown |
| rs7722615 | 5 | 7989257 | T | 0.11 | *MTRR* | function unknown |
| rs2174147 | 5 | 7996261 | A | 0.35 | *MTRR* | function unknown |
| rs2961994 | 5 | 8026017 | G | 0.11 | *MTRR* | function unknown |
| rs12523589 | 5 | 8027851 | T | 0.44 | *MTRR* | function unknown |
| rs13158739 | 5 | 8029718 | G | 0.05 | *MTRR* | function unknown |
| rs1377489 | 5 | 8036385 | C | 0.23 | *MTRR* | function unknown |
| rs13162612 | 5 | 8043021 | G | 0.06 | *MTRR* | function unknown |
| rs876712 | 5 | 8047543 | A | 0.15 | *MTRR* | function unknown |
| rs2924471 | 5 | 8055261 | C | 0.13 | *MTRR* | function unknown |
| rs3752857 | 5 | 8057798 | C | 0.38 | *MTRR* | function unknown |
| rs6886541 | 5 | 8062672 | C | 0.42 | *MTRR* | function unknown |
| rs6860289 | 5 | 8062748 | A | 0.23 | *MTRR* | function unknown |
| rs1470866 | 5 | 8069278 | T | 0.35 | *MTRR* | function unknown |
| rs2961986 | 5 | 8073071 | A | 0.37 | *MTRR* | function unknown |
| rs10475407 | 5 | 8093077 | C | 0.34 | *MTRR* | function unknown |
| rs7716010 | 5 | 8100893 | A | 0.30 | *MTRR* | function unknown |
| rs6860481 | 5 | 8113536 | C | 0.25 | *MTRR* | function unknown |
| rs1506105 | 5 | 8121656 | C | 0.45 | *MTRR* | function unknown |
| rs10057537 | 5 | 8123994 | G | 0.27 | *MTRR* | function unknown |
| rs6555523 | 5 | 8130362 | A | 0.13 | *MTRR* | function unknown |
| rs13172069 | 5 | 8131880 | C | 0.18 | *MTRR* | function unknown |
| rs2658161 | 5 | 8133694 | C | 0.05 | *MTRR* | function unknown |
| rs731099 | 5 | 8143945 | C | 0.09 | *MTRR* | function unknown |
| rs336164 | 5 | 8149540 | C | 0.40 | *MTRR* | function unknown |
| rs10512943 | 5 | 8151979 | G | 0.18 | *MTRR* | function unknown |
| rs1565198 | 5 | 8155254 | C | 0.49 | *MTRR* | function unknown |
| rs336178 | 5 | 8173436 | T | 0.11 | *MTRR* | function unknown |
| rs13170530 | 5 | 8174188 | C | 0.20 | *MTRR* | function unknown |
| rs901893 | 5 | 8175049 | C | 0.38 | *MTRR* | function unknown |
| rs336174 | 5 | 8177920 | A | 0.25 | *MTRR* | function unknown |
| rs1823809 | 5 | 8188341 | G | 0.33 | *MTRR* | function unknown |
| rs7702422 | 5 | 8188395 | C | 0.30 | *MTRR* | function unknown |
| rs7707765 | 5 | 8189392 | T | 0.38 | *MTRR* | function unknown |
| rs11134290 | 5 | 8202536 | T | 0.12 | *MTRR* | function unknown |
| rs3843472 | 5 | 8206385 | G | 0.13 | *MTRR* | function unknown |
| rs6893114 | 5 | 8212115 | T | 0.25 | *MTRR* | function unknown |
| rs6892570 | 5 | 8226339 | G | 0.42 | *MTRR* | function unknown |
| rs10512947 | 5 | 8231744 | C | 0.19 | *MTRR* | function unknown |
| rs10512948 | 5 | 8233351 | C | 0.21 | *MTRR* | function unknown |
| rs1911009 | 5 | 8241011 | C | 0.43 | *MTRR* | function unknown |
| rs4639181 | 5 | 8243414 | T | 0.10 | *MTRR* | function unknown |
| rs2129952 | 5 | 8248078 | G | 0.33 | *MTRR* | function unknown |
| rs6880502 | 5 | 8259322 | C | 0.30 | *MTRR* | function unknown |
| rs906087 | 5 | 8270536 | C | 0.05 | *MTRR* | function unknown |
| rs6555540 | 5 | 8303435 | T | 0.22 | *MTRR* | function unknown |
| rs1189843 | 5 | 8319632 | A | 0.23 | *MTRR* | function unknown |
| rs925203 | 5 | 8328783 | T | 0.30 | *MTRR* | function unknown |
| rs2940556 | 5 | 8349784 | C | 0.45 | *MTRR* | function unknown |
| rs340664 | 5 | 8363456 | A | 0.45 | *MTRR* | function unknown |
| rs446249 | 5 | 8376813 | T | 0.27 | *MTRR* | function unknown |
| rs340680 | 5 | 8385180 | A | 0.19 | *MTRR* | function unknown |
| rs182905 | 5 | 8395369 | T | 0.20 | *MTRR* | function unknown |
| rs11743533 | 5 | 8404594 | A | 0.09 | *MTRR* | function unknown |
| rs4702547 | 5 | 8412204 | G | 0.40 | *MTRR* | function unknown |
| rs340698 | 5 | 8422650 | C | 0.27 | *MTRR* | function unknown |
| rs10512960 | 5 | 8439926 | G | 0.05 | *MTRR* | function unknown |
| rs9313239 | 5 | 8459533 | T | 0.36 | *MTRR* | function unknown |
| rs11954087 | 5 | 8466223 | C | 0.16 | *MTRR* | function unknown |
| rs341893 | 5 | 8467900 | A | 0.26 | *MTRR* | function unknown |
| rs921186 | 5 | 8468123 | C | 0.13 | *MTRR* | function unknown |
| rs9902011 | 17 | 18231652 | C | 0.05 | *SHMT1* | utr-variant-3-prime, downstream-variant-500B |
| rs12952556 | 17 | 18231797 | C | 0.25 | *SHMT1* | utr-variant-3-prime, downstream-variant-500B |
| rs1979276 | 17 | 18231998 | A | 0.32 | *SHMT1* | utr-variant-3-prime |
| rs3783 | 17 | 18232017 | C | 0.20 | *SHMT1* | utr-variant-3-prime |
| rs1979277 | 17 | 18232096 | A | 0.25 | *SHMT1* | missense |
| rs6502648 | 17 | 18232571 | A | 0.05 | *SHMT1* | intron-variant |
| rs2125154 | 17 | 18232958 | G | 0.05 | *SHMT1* | intron-variant |
| rs12937300 | 17 | 18233810 | C | 0.20 | *SHMT1* | intron-variant |
| rs8080285 | 17 | 18234028 | G | 0.07 | *SHMT1* | intron-variant |
| rs4925174 | 17 | 18234467 | G | 0.03 | *SHMT1* | intron-variant |
| rs7215491 | 17 | 18236042 | C | 0.07 | *SHMT1* | intron-variant |
| rs2273028 | 17 | 18239012 | A | 0.29 | *SHMT1* | intron-variant |
| rs2168781 | 17 | 18240746 | G | 0.44 | *SHMT1* | intron-variant |
| rs4924848 | 17 | 18242443 | T | 0.05 | *SHMT1* | intron-variant |
| rs9897954 | 17 | 18243638 | A | 0.03 | *SHMT1* | intron-variant |
| rs9904323 | 17 | 18244293 | G | 0.03 | *SHMT1* | intron-variant |
| rs11868708 | 17 | 18244613 | C | 0.24 | *SHMT1* | intron-variant |
| rs4924849 | 17 | 18247280 | T | 0.24 | *SHMT1* | intron-variant |
| rs9909104 | 17 | 18248021 | C | 0.31 | *SHMT1* | intron-variant |
| rs9902498 | 17 | 18249446 | T | 0.03 | *SHMT1* | intron-variant |
| rs2273027 | 17 | 18250527 | T | 0.43 | *SHMT1* | intron-variant |
| rs4925177 | 17 | 18252868 | G | 0.27 | *SHMT1* | intron-variant |
| rs4500798 | 17 | 18255079 | G | 0.27 | *SHMT1* | intron-variant |
| rs2273026 | 17 | 18256979 | T | 0.13 | *SHMT1* | intron-variant |
| rs4924750 | 17 | 18260753 | G | 0.19 | *SHMT1* | intron-variant |
| rs669340 | 17 | 18263957 | G | 0.36 | *SHMT1* | intron-variant |
| rs2461837 | 17 | 18265467 | T | 0.20 | *SHMT1* | intron-variant |
| rs9900382 | 17 | 18266532 | T | 0.03 | *SHMT1* | intron-variant |
| rs8065874 | 17 | 18275847 | T | 0.41 | *SHMT1* | unknown |
| rs2853533 | 18 | 658064 | C | 0.30 | *C18orf56, TYMS* | intron-variant, missense |
| rs502396 | 18 | 659236 | T | 0.41 | *C18orf56, TYMS* | intron-variant, upstream-variant-2KB |
| rs2847153 | 18 | 661647 | A | 0.27 | *TYMS* | intron-variant |
| rs1001761 | 18 | 662103 | G | 0.40 | *TYMS* | intron-variant |
| rs2612095 | 18 | 662370 | T | 0.40 | *TYMS* | intron-variant |
| rs15872 | 18 | 668258 | T | 0.48 | *TYMS* | intron-variant |
| rs2847150 | 18 | 668300 | A | 0.48 | *TYMS* | intron-variant |
| rs16948305 | 18 | 668465 | T | 0.09 | *TYMS* | intron-variant |
| rs2853528 | 18 | 668650 | T | 0.48 | *TYMS* | intron-variant |
| rs2853532 | 18 | 670414 | T | 0.48 | *TYMS* | utr-variant-3-prime, intron-variant |
| rs699517 | 18 | 673016 | T | 0.48 | *TYMS* | utr-variant-3-prime |
| rs706209 | 21 | 44473425 | A | 0.40 | *CBS* | utr-variant-3-prime |
| rs706208 | 21 | 44473446 | G | 0.40 | *CBS* | utr-variant-3-prime |
| rs12613 | 21 | 44473691 | T | 0.08 | *CBS* | utr-variant-3-prime |
| rs1051319 | 21 | 44473867 | C | 0.21 | *CBS* | utr-variant-3-prime, intron-variant |
| rs2124458 | 21 | 44475680 | C | 0.34 | *CBS* | intron-variant |
| rs2124459 | 21 | 44475714 | C | 0.46 | *CBS* | intron-variant |
| rs234702 | 21 | 44477543 | G | 0.13 | *CBS* | intron-variant |
| rs6586281 | 21 | 44478393 | A | 0.14 | *CBS* | intron-variant |
| rs9325622 | 21 | 44479701 | G | 0.35 | *CBS* | intron-variant |
| rs11203172 | 21 | 44480115 | T | 0.08 | *CBS* | intron-variant |
| rs4920037 | 21 | 44481891 | A | 0.14 | *CBS* | intron-variant |
| rs1789953 | 21 | 44482936 | T | 0.13 | *CBS* | intron-variant |
| rs12329764 | 21 | 44483248 | A | 0.10 | *CBS* | intron-variant |
| rs234705 | 21 | 44483772 | T | 0.22 | *CBS* | intron-variant |
| rs9974224 | 21 | 44484742 | T | 0.08 | *CBS* | intron-variant |
| rs234706 | 21 | 44485350 | A | 0.22 | *CBS* | synonymous-codon |
| rs234709 | 21 | 44486964 | T | 0.43 | *CBS* | intron-variant |
| rs234711 | 21 | 44487111 | A | 0.14 | *CBS* | intron-variant |
| rs2851391 | 21 | 44487404 | T | 0.40 | *CBS* | intron-variant |
| rs234712 | 21 | 44487453 | A | 0.08 | *CBS* | intron-variant |
| rs234715 | 21 | 44488395 | T | 0.14 | *CBS* | intron-variant |
| rs9982015 | 21 | 44490092 | C | 0.08 | *CBS* | intron-variant |
| rs11701048 | 21 | 44491425 | T | 0.09 | *CBS* | intron-variant |
| rs1788484 | 21 | 44496941 | T | 0.23 | *CBS* | upstream-variant-2KB |

^a^ Data source: NCBI Genome Reference Consortium Human Build 37 patch release 5 (GRCh37.p5)

^b^ SNPs located within 500kb of given gene
