# Supplementary material for: Investigating Multiple Candidate Genes and Nutrients in the Folate Metabolism Pathway to Detect Genetic and Nutritional Risk Factors for Lung Cancer
Source: PLoS One. 2013 Jan 23;8(1):e53475. doi: 10.1371/journal.pone.0053475 (PMC3553105; doi:10.1371/journal.pone.0053475)
Supplement: Table S2 — SNPs selected from initial screening stratified by smoking status. Lists all snps that passed the first screen of association using PPI greater than 0.35 for each smoking status. (DOCX) [file pone.0053475.s002.docx]

**Supplementary Table S2: SNPs Selected from Initial Screening Stratified by Smoking Status**^a^

| **Model** | **SNP** | **Gene**^b^ | **PPI**^c^ |
| --- | --- | --- | --- |
| **Current Smokers** | rs234702 | *CBS* | 0.87 |
|  | rs1051319 | *CBS* | 0.48 |
|  | rs2174147 | *MTRR* | 0.39 |
|  | rs2658161 | *MTRR* | 0.41 |
|  | rs6893114 | *MTRR* | 0.84 |
|  | rs906087 | *MTRR* | 0.37 |
|  | rs2940556 | *MTRR* | 0.48 |
|  | rs921186 | *MTRR* | 0.52 |
|  | Rs7730643 | *MTRR* | 0.41 |
|  | rs11868708 | *SHMT1* | 0.39 |
|  | rs2273026 | *SHMT1* | 0.43 |
|  | rs16948305 | *TYMS* | 0.65 |
| **Former Smokers** | rs12613 | *CBS* | 0.4 |
|  | rs234702 | *CBS* | 0.44 |
|  | rs9974224 | *CBS* | 0.74 |
|  | rs17375901 | *MTHFR* | 0.57 |
|  | rs2184226 | *MTHFR* | 0.42 |
|  | rs4659723 | *MTR* | 0.36 |
|  | rs876712 | *MTRR* | 0.51 |
|  | rs2658161 | *MTRR* | 0.37 |
|  | rs13170530 | *MTRR* | 0.5 |
|  | rs1823809 | *MTRR* | 0.6 |
|  | rs11134290 | *MTRR* | 0.35 |
|  | rs2129952 | *MTRR* | 0.49 |
|  | rs446249 | *MTRR* | 0.53 |
|  | rs16948305 | *TYMS* | 0.59 |
| **Never Smokers** | rs706209 | *CBS* | 0.39 |
|  | rs12613 | *CBS* | 0.44 |
|  | rs2124458 | *CBS* | 0.47 |
|  | rs6586281 | *CBS* | 0.64 |
|  | rs6696752 | *MTHFR* | 0.52 |
|  | rs2184226 | *MTHFR* | 0.68 |
|  | rs12121543 | *MTHFR* | 0.56 |
|  | rs2066462 | *MTHFR* | 0.63 |
|  | rs1801133 | *MTHFR C677T* | 0.47 |
|  | rs13306567 | *MTHFR* | 0.37 |
|  | rs17037390 | *MTHFR* | 0.37 |
|  | rs9651118 | *MTHFR* | 0.58 |
|  | rs7730643 | *MTRR* | 0.69 |
|  | rs3776467 | *MTRR* | 0.36 |
|  | rs2174147 | *MTRR* | 0.36 |
|  | rs2961994 | *MTRR* | 0.58 |
|  | rs13162612 | *MTRR* | 0.94 |
|  | rs876712 | *MTRR* | 0.38 |
|  | rs2924471 | *MTRR* | 0.96 |
|  | rs10475407 | *MTRR* | 0.47 |
|  | rs731099 | *MTRR* | 0.39 |
|  | rs11134290 | *MTRR* | 0.49 |
|  | rs10512948 | *MTRR* | 0.47 |
|  | rs2168781 | *SHMT1* | 0.43 |
|  | rs2853533 | *TYMS* | 0.44 |
|  | rs16948305 | *TYMS* | 0.46 |

^a^ without nutrition variables

^b^ SNPs located within 500kb of given gene

^c^ PPI = Posterior probability of inclusion
