# Supplementary material for: Investigating Multiple Candidate Genes and Nutrients in the Folate Metabolism Pathway to Detect Genetic and Nutritional Risk Factors for Lung Cancer
Source: PLoS One. 2013 Jan 23;8(1):e53475. doi: 10.1371/journal.pone.0053475 (PMC3553105; doi:10.1371/journal.pone.0053475)
Supplement: Table S3 — Nutrients Selected from Initial Screening Stratified by Smoking Status. Table listing nutrients that passed the first screen of association using PPI greater than 0.35, for each smoking status. (DOCX) [file pone.0053475.s003.docx]

**Supplementary Table S3: Nutrients Selected from Initial Screening Stratified by Smoking Status**^a^

| **Model** | **Nutrients** | **PPI**^b^ |
| --- | --- | --- |
| **Current Smokers** | Alcohol | 0.76 |
|  | Vitamin B_6_ | 0.56 |
| **Former Smokers** | Alcohol | 0.88 |
|  | Carbohydrate | 0.61 |
|  | Protein | 0.44 |
|  | Betaine | 0.54 |
|  | Methionine | 0.37 |
|  | Thiamin | 0.36 |
|  | Vitamin B_12_ | 0.39 |
| **Never Smokers** | Carbohydrate | 0.52 |
|  | Protein | 0.38 |
|  | Choline | 0.35 |
|  | Folate | 0.39 |
|  | Riboflavin | 0.37 |
|  | Thiamin | 0.41 |

^a^ without SNPs

^b^ PPI = Posterior Probability of Inclusion
