# Supplementary material for: Investigating Multiple Candidate Genes and Nutrients in the Folate Metabolism Pathway to Detect Genetic and Nutritional Risk Factors for Lung Cancer
Source: PLoS One. 2013 Jan 23;8(1):e53475. doi: 10.1371/journal.pone.0053475 (PMC3553105; doi:10.1371/journal.pone.0053475)
Supplement: Table S4 — Linkage Disequilibrium Magnitudes Between SNPs in the Final Model. Table listing the absolute LD magnitudes for thos SNPs identified in any final model. (DOCX) [file pone.0053475.s004.docx]

Supplementary Table S4: Linkage Disequilibrium Magnitudes Between SNPs in the Final Model

| **SNP ID** | **rs9651118** | **rs13162612** | **rs876712** | **rs2924471** | **rs10475407** | **rs2658161** | **rs13170530** | **rs11134290** | **rs6893114** | **rs10512948** | **rs16948305** |
| --- | --- | --- | --- | --- | --- | --- | --- | --- | --- | --- | --- |
| **rs9651118** | 1 | 0 | 0 | 0 | 0 | 0 | 0 | 0 | 0 | 0 | 0 |
| **rs13162612** |  | 1 | 0.007 | 0.561 | 0.037 | 0 | 0.003 | 0.018 | 0.004 | 0.015 | 0 |
| **rs876712** |  |  | 1 | 0.008 | 0.022 | 0.002 | 0 | 0.135 | 0.007 | 0.014 | 0 |
| **rs2924471** |  |  |  | 1 | 0.069 | 0.006 | 0.003 | 0.006 | 0.002 | 0.009 | 0 |
| **rs10475407** |  |  |  |  | 1 | 0.034 | 0 | 0.003 | 0.048 | 0.039 | 0 |
| **rs2658161** |  |  |  |  |  | 1 | 0.021 | 0.378 | 0.001 | 0.009 | 0 |
| **rs13170530** |  |  |  |  |  |  | 1 | 0.026 | 0.036 | 0.02 | 0 |
| **rs11134290** |  |  |  |  |  |  |  | 1 | 0.012 | 0.022 | 0 |
| **rs6893114** |  |  |  |  |  |  |  |  | 1 | 0.625 | 0 |
| **rs10512948** |  |  |  |  |  |  |  |  |  | 1 | 0 |
| **rs16948305** |  |  |  |  |  |  |  |  |  |  | 1 |
