# Supplementary material for: Investigating Multiple Candidate Genes and Nutrients in the Folate Metabolism Pathway to Detect Genetic and Nutritional Risk Factors for Lung Cancer
Source: PLoS One. 2013 Jan 23;8(1):e53475. doi: 10.1371/journal.pone.0053475 (PMC3553105; doi:10.1371/journal.pone.0053475)
Supplement: Table S5 — Correlation Between Nutrients in Final Model. Table listing the correlation between nutrients identified in any final model. (DOCX) [file pone.0053475.s005.docx]

Supplementary Table S5: Correlation Between Nutrients in Final Model

| **Nutrients** | **Betaine** | **Choline** | **Riboflavin** |
| --- | --- | --- | --- |
| **Betaine** | 1.000000 | 0.613660 | 0.307124 |
| **Choline** |  | 1.000000 | 0.403982 |
| **Riboflavin** |  |  | 1.000000 |
